# Eomesodermin expression in CD4^+^T‐cells associated with disease progression in amyotrophic lateral sclerosis

**DOI:** 10.1111/cns.14503

**Published:** 2023-10-18

**Authors:** Sheng Chen, Xiao Huan, Chun‐Zuan Xu, Su‐Shan Luo, Chong‐Bo Zhao, Hua‐Hua Zhong, Xue‐Ying Zheng, Kai Qiao, Yi Dong, Ying Wang, Chang‐Yun Liu, Hua‐Pin Huang, Yan Chen, Zhang‐Yu Zou

**Affiliations:** ^1^ Department of Neurology Fujian Medical University Union Hospital Fuzhou China; ^2^ Institute of Clinical Neurology Fujian Medical University Fuzhou China; ^3^ Huashan Rare Disease Center and Department of Neurology Huashan Hospital, Shanghai Medical College, National Center for Neurological Disorders, Fudan University Shanghai China; ^4^ Department of Biostatistics, School of Public Health and Key Laboratory of Public Health Safety Fudan University Shanghai China; ^5^ Department of Pharmacy Fudan University Huashan Hospital Shanghai China

**Keywords:** amyotrophic lateral sclerosis, bulbar onset, CD4^+^T lymphocyte, CXCR3, eomesodermin, neurofilament light chain, prognosis

## Abstract

**Aim:**

To clarify the role of Eomesodermin (EOMES) to serve as a disease‐relevant biomarker and the intracellular molecules underlying the immunophenotype shifting of CD4^+^T subsets in amyotrophic lateral sclerosis (ALS).

**Methods:**

The derivation and validation cohorts included a total of 148 ALS patients and 101 healthy controls (HCs). Clinical data and peripheral blood were collected. T‐cell subsets and the EOMES expression were quantified using multicolor flow cytometry. Serum neurofilament light chain (NFL) was measured. In 1‐year longitudinal follow‐ups, the ALSFRS‐R scores and primary endpoint events were further recorded in the ALS patients of the validation cohort.

**Results:**

In the derivation cohort, the CD4^+^EOMES^+^T‐cell subsets were significantly increased (*p* < 0.001). EOMES^+^ subset was positively correlated with increased serum NFL levels in patients with onset longer than 12 months. In the validation cohort, the elevated CD4^+^EOMES^+^T‐cell proportions and their association with NFL levels were also identified. The longitudinal study revealed that ALS patients with higher EOMES expression were associated with higher progression rates (*p* = .010) and worse prognosis (*p* = .003).

**Conclusions:**

We demonstrated that increased CD4^+^EOMES^+^T‐cell subsets in ALS were associated with disease progression and poor prognosis. Identifying these associations may contribute to a better understanding of the immunopathological mechanism of ALS.

## Graphical abstract

Eomesodermin was highly expressed in the peripheral CD4^+^T‐cells derived from ALS patients. Elevated CXCR3 expression in CD4^+^EOMES^+^ T‐cells differentiates ALS from HCs. EOMES^+^ subsets were correlated with serum NFL levels. ALS patients with higher EOMES expression were associated with higher progression rates and worse prognosis.

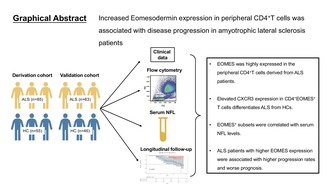

## 1 INTRODUCTION

Amyotrophic lateral sclerosis (ALS) is a devastating neurodegenerative disease characterized by progressive loss of upper and lower motor neurons that leads to paralysis, dysphagia, dysarthria, and eventually respiratory failure.^1^ Multiple factors may contribute to ALS susceptibility. Among them, neuroinflammation is one of the most important hallmarks and potential therapeutic targets of ALS.^2^

It has been well documented that ALS patients have persistent peripheral inflammation, which may exacerbate the neurodegenerative process. Among them, the CD4^+^T‐cell counts were considered to play a critical role. CD4^+^T‐cells were significantly reduced in a cohort of 284 ALS patients from West China,^3^ as well as in other reports from Australia and the United States.^4^, ^5^ Meanwhile, infiltrated T‐cells have been identified in the spinal cord, the precentral gyrus, and the degenerating corticospinal tract in autopsy studies.^6^, ^7^, ^8^ Elimination of the peripheral CD4^+^T‐cells in the transgenic mice accelerated the clinical severity.^9^, ^10^ A population‐based longitudinal cohort study recently demonstrated that several altered T‐cell subpopulations were related to the risk of death in ALS patients.^11^ However, the intracellular molecules underlying the phenotype shifting in CD4^+^T‐cells among ALS remain largely unelucidated.

Eomesodermin (EOMES), a transcription factor, was closely linked to the lineage differentiation of CD4^+^T subsets.^12^, ^13^, ^14^, ^15^ The EOMES expression in CD4^+^T‐cells has been reported to be essential in the induction of chronic neuroinflammation.^16^ More recently, elevated EOMES‐positive T‐cells were demonstrated in patients with secondary progressive multiple sclerosis (SPMS), a central nervous system (CNS) demyelinating disease with neurodegenerative pathogenesis. The EOMES‐positive T subsets were significantly associated with the disease progression and were able to predict the disease worsening with 80% accuracy.^17^ More intriguingly, a recent study has noticed a substantially increased proportion of EOMES‐expressing cells in the cerebrospinal fluid (CSF) expanded T‐cells of ALS patients.^18^ Although multiple pieces of evidence have hinted at a potential role of EOMES in the pathogenesis of ALS, the EOMES expression in CD4^+^T‐cells among ALS patients and its potential influence on the disease progression have not been clarified thoroughly.

Thus, we hypothesized that the EOMES expression in CD4^+^T‐cells was potentially associated with the CD4^+^T phenotype shifting and the disease progression among ALS patients. In the current study, we used multicolor flow cytometry to measure the changes in EOMES expression among peripheral CD4^+^T subsets and assessed the associations between these subsets with disease progression and serum neurofilament light chain (NFL) in a cohort with ALS.

## 2 METHODS

### 2.1 Study population

The current study included a derivation cohort and a validation cohort with a total of 249 participants recruited from two tertiary hospitals located in East and South China. The derivation cohort was comprised of 85 ALS patients and 55 age‐ and gender‐matched healthy controls (HCs), who were recruited from two independent neuromuscular referral centers in Fudan University Huashan Hospital and Fujian Medical University Union Hospital, from September 13, 2018, to June 1, 2021. To further validate our results, a validation cohort, including 63 ALS patients and 46 HCs, was recruited from May 16, 2022, to October 19, 2022. The diagnosis of ALS was made by at least two experienced neurologists according to the revised EI Escorial criteria.^19^ The Revised Amyotrophic Lateral Sclerosis Functional Rating Scale (ALSFRS‐R) was used for clinical assessment. King's staging system^20^ and ALS‐Milano Torino functional staging system (ALS‐MiToS)^21^ were used for patient grading. Exclusion criteria for both patients and controls were acute or chronic infection, a positive family history of ALS, a previous diagnosis of neurodegenerative disorders, autoimmune diseases, or immunomodulatory medication at blood sampling. In 1‐year longitudinal follow‐ups, the ALSFRS‐R scores and primary endpoint events (including invasive mechanical ventilation, using noninvasive mechanical ventilation over 22 h per day, and death) were further recorded in the validation cohort recruited from Fujian Medical University Union Hospital. A flowchart displaying the study design is presented in Figure S1. The study protocol was approved by the ethics committees of Fudan University Huashan Hospital (2019‐603) and Fujian Medical University Union Hospital (2019GZR032). Written informed consent was obtained from each participant.

### 2.2 Blood sampling and flow cytometry

Peripheral blood was collected using a BD Vacutainer® blood collection tube containing EDTA and immediately sent for flow cytometry analysis. For flow cytometry, a 200‐μL aliquot of whole blood was stained with a cocktail of surface markers, which was selected mainly derived from the standardized phenotyping panel by the Human Immunophenotyping Consortium^22^ (the antibodies included anti‐CD3, anti‐CD4, anti‐CXCR3, anti‐CCR4, anti‐CCR6, anti‐CCR7, anti‐CD45RA, anti‐CD25, and anti‐CD127). Then, the mixture was lysed using red blood cell lysing buffer (TIANGEN). Subsequently, cells were fixed and permeabilized using eBioscience Foxp3/Transcription Factor Buffer Set (Thermo Fisher Scientific) and stained with anti‐EOMES antibody (clone: WD1928; Thermo Fisher Scientific) according to the manufacturer's instructions. Appropriate fluorescein‐conjugated, isotype‐matched, irrelevant antibodies were used as isotype controls. The flow cytometry experiments of the derivation cohort were performed using Attune NxT Flow Cytometer (Thermo Fisher Scientific) and analyzed on FlowJo VX Software (FlowJo, LLC). The flow cytometry experiments of the validation cohort were performed using BD FACSCelesta™ Flow Cytometer (BD Life Sciences).

### 2.3 The CD4^+^T subset gating strategies

The CD4^+^T subset gating was performed according to the previous reports.^22^, ^23^, ^24^ T‐cell populations were observed in the derivation cohort, including (1) counts of CD3^+^ and CD4^+^ cells; (2) the proportions of conventional CD4^+^ subsets (%) based on the expression of CXCR3, CCR4, CCR6, CD25, and CD127 (i.e., Th1, Th2, Th9, Th17, Th17.1, and Treg); (3) the proportions of effector memory subsets (%) within CD4^+^T‐cells based on the expression of CCR7 and CD45RA (i.e., CD45RA‐positive effector memory T [TEMRA], effector memory T [TEM], central memory T [TCM], and naive T [TNAIVE]); (4) the proportions of EOMES in the CD4^+^ subsets (%) mentioned above; (5) the proportions of unconventional CD4^+^ subsets (%) (CD3^+^CD4^+^CXCR3^+^EOMES^+^ subset). The percentage of EOMES in the CD4^+^ T‐cells, as well as the conventional CD4 subsets, and the CD3^+^CD4^+^CXCR3^+^EOMES^+^ subsets, were measured in the validation cohort.

### 2.4 Serum NFL quantification

Serum NFL levels were quantified with NFL assay kits (Cat No: 103186; Quanterix) on the ultrasensitive single‐molecule array (SIMOA) platform according to the manufacturer's instruction. Calibrators and quality controls were measured in duplicates. All sample measurements were diluted at a 1:4 ratio and performed on a single‐run basis. Operators were blinded to the participants' disease status.

### 2.5 Statistical analyses

Qualitative variables were compared by the chi‐square or Fisher's exact test. Quantitative variables for the ALS patients and HCs were summarized with descriptive statistics, such as mean, standard deviation (SD), median, interquartile range (IQR), and proportions. For CD4^+^T subsets, proportions less than 0.05% were not taken into statistical analysis. Mann–Whitney tests were performed for comparisons. A receiver operating characteristic (ROC) curve was generated, and the area under the curve (AUC) was calculated to assess the performance of CD4^+^T subsets. The Spearman correlation was used in assessing the associations among the patient's functional status, serum NFL, and immune biomarkers. To explore the association between the EOMES expression and the risk of developing endpoint events, multiple variate COX regression models were adopted and survival curves were constructed. Log‐rank test was then applied to assess the differences between the survival curves. Statistical analyses and visualizations were performed using GraphPad Prism version 9 and R version 4.2.2. *p*‐Values were two‐tailed with a significant level of 0.05.

## 3 RESULTS

### 3.1 Clinical and demographic features of the study population

The clinical and demographic features are summarized in Table 1. In the derivation cohort, the ALS group included 85 patients (age 57.1 [SD 12.1] years, male 62.2% [52/85], bulbar onset 17.7% [15/85]), whereas the HCs included 55 participants (age 53.0 [SD 12.7] years, male 54.5% [30/55]). The median disease duration was 12 months (IQR: 8.0–18.0). The median ALSFRS‐R score at blood sampling was 41 (IQR: 34–43). The median decrease of ALSFRS‐R (ΔALSFRS‐R) was estimated at 7 (IQR: 5–14), with an average disease progression rate (ΔALSFRS‐R/month) at 0.6 (IQR: 0.4–1.2). Clinical status in the ALS cohort classified by King's staging was as follows: stage 2 (65.9%), including 19 patients in stage 2A and 37 patients in stage 2B, stage 3 (27.0%, 23/85), stage 4 (5.9%, 5/85), and stage 5 (1.2%, 1/85). For ALS‐MiToS staging, the proportions for each stage were as follows: 52.9% (45/85) at stage 0, 29.4% (25/85) at stage 1, 11.8% (10/85) at stage 2, 3.5% (3/85) at stage 3, 1.2% (1/85) at stage 4, and 1.2% (1/85) at stage 5.

**TABLE 1:** Clinical and demographic features of the study population.

	Primary cohort			Validation cohort		
	ALS	HC	*p*	ALS	HC	*p*
*n*	85	55		63	46	
Male/Female, Age	52/33	30/25	0.437	33/30	27/19	0.562
(Mean ± SD, years)	57.1 ± 12.1	53.0 ± 12.7	0.055	56.5 ± 9.0	53.4 ± 10.4	0.106
Site of onset, *N* (%)						
Bulbar	15 (17.7)	—		24 (38.1)	—	
Upper limbs	33 (38.8)	—		25 (39.7)	—	
Lower limbs	37 (43.5)	—		14 (22.2)	—	
Disease duration (Median [IQR], months)	12.0 [8.0–18.0]	—		11.0 [6.0–19.0]	—	
ALSFRS‐R (first visit) (Median [IQR])	41 [34–43]	—		40 [37–43]	—	
ΔALSFRS‐R/month (Median [IQR])	0.6 [0.4–1.2]	—		0.7 [0.3–1.2]	—	
King's Staging, *N* (%)						
2A	19 (22.4%)			16 (25.4%)		
2B	37 (43.5%)			28 (44.5%)		
3	23 (27.0%)			14 (22.2%)		
4	5 (5.9%)			5 (7.9%)		
5	1 (1.2%)			0 (0%)		
ALS‐MiToS staging, *N* (%)						
0	45 (52.9%)	—		51 (80.9%)	—	
1	25 (29.4%)			7 (11.1%)		
2	10 (11.8%)			3 (4.8%)		
3	3 (3.5%)			2 (3.2%)		
4	1 (1.2%)			0 (0%)		
5	1 (1.2%)			0 (0%)		

Abbreviations: ALS, amyotrophic lateral sclerosis; ALSFRS‐R, The Revised Amyotrophic Lateral Sclerosis Functional Rating Scale; ALS‐MiToS, ALS‐Milano Torino functional staging; HC, healthy control; IQR, interquartile range; SD, standard deviation.

In the validation cohort, the ALS group included 63 patients (age 56.5 [SD 9.0] years, male 52.4% [33/63], bulbar onset 38.1% [24/63]), whereas the healthy controls included 46 participants (age 53.4 [SD 10.4] years, male 58.7% [27/46]). The median disease duration was 11 months (IQR: 6.0–19.0). The median ALSFRS‐R score at blood sampling was 40 (IQR: 37–43). The average disease progression rate (ΔALSFRS‐R/month) was 0.7 (IQR: 0.3–1.2). Clinical status in the ALS cohort classified by King's staging was as follows: stage 2 (69.9%), including 16 patients in stage 2A and 28 patients in stage 2B, stage 3 (22.2%, 14/63), and stage 4 (7.9%, 5/63). For ALS‐MiToS staging, the proportions for each stage were as follows: 80.9% (51/63) at stage 0, 11.1% (7/63) at stage 1, 4.8% (3/63) at stage 2, and 3.2% (2/63) at stage 3.

### 3.2 EOMES was highly expressed in the peripheral CD4^+^T‐cells derived from ALS patients

The frequencies of CD4^+^T subsets are summarized in Table 2. In the derivation cohort, CD3^+^T and CD4^+^T‐cell counts per 10^6^/mL blood were both significantly decreased in ALS patients (0.29 × 10^6^/mL [IQR: 0.15–0.41] vs. 0.47 × 10^6^/mL [IQR: 0.26–0.56], *p <* 0.001, and 0.15 × 10^6^/mL [IQR: 0.08–0.22] vs. 0.27 × 10^6^/mL [IQR: 0.15–0.34], *p <* 0.001, respectively). Immunophenotyping of the conventional CD4^+^T subsets revealed increased Th1 proportions in ALS patients (12.4 [IQR: 9.7–16.5] vs. 9.1 [IQR: 6.8–13.8], *p <* 0.001). Additionally, there was a trend in increased TEMRA and TEM proportions (3.2 [IQR: 1.8–6.2] vs. 2.0 [IQR: 1.2–5.7], *p* = 0.072, and 23.6 [IQR: 17.6–32.8] vs. 20.0 [IQR: 13.6–28.6], *p* = 0.116), and decreased TCM proportions (43.3 [IQR: 34.1–54.6] vs. 48.6 [IQR: 41.8–54.8], *p* = 0.222) in ALS cohort compared to that in healthy controls (albeit not significant). No significant changes were noticed in other CD4^+^T subsets.

**TABLE 2:** Comparison of conventional subsets and EOMES expression between groups.

	ALS (Median [IQR])	HC (Median [IQR])	*p*
Derivation cohort			
CD3^+^T counts (per 10^6^/mL blood)	0.29 [0.15–0.41]	0.47 [0.26–0.56]	<0.001
CD4^+^T counts (per 10^6^/mL blood)	0.15 [0.08–0.22]	0.27 [0.15–0.34]	<0.001
CD3^+^T (% in PBMC)	61.9 [53.9–70.2]	63.3 [57.2–74.7]	0.287
CD4^+^T (% in CD3^+^T)	32.4 [26.6–39.4]	36.3 [28.7–44.7]	0.023
Th1 (% in CD4^+^T)	12.4 [9.7–16.5]	9.1 [6.8–13.8]	<0.001
Th2 (% in CD4^+^T)	16.1 [10.8–22.8]	15.1 [10.4–22.6]	0.676
Th9 (% in CD4^+^T)	7.4 [5.8–10.7]	7.8 [5.0–10.0]	0.719
Th17 (% in CD4^+^T)	9.6 [7.4–14.2]	10.0 [7.3–13.7]	0.845
Th17.1 (% in CD4^+^T)	5.4 [3.2–7.8]	4.6 [3.2–7.6]	0.520
Treg (% in CD4^+^T)	7.2 [6.1–8.4]	7.7 [6.3–8.9]	0.332
TEMRA (% in CD4^+^T)	3.2 [1.8–6.2]	2.0 [1.2–5.7]	0.072
TEM (% in CD4^+^T)	23.6 [17.6–32.8]	20.0 [13.6–28.6]	0.116
TCM (% in CD4^+^T)	43.3 [34.1–54.6]	48.6 [41.8–54.8]	0.222
TNAIVE (% in CD4^+^T)	24.1 [14.8–33.3]	24.2 [16.3–33.6]	0.313
EOMES (% in CD4^+^T)	12.7 [8.7–18.7]	7.2 [5.0–13.7]	<0.001
EOMES in Th1	23.2 [12.2–36.0]	15.3 [10.2–26.2]	0.010
EOMES in Th2	3.2 [1.6–6.2]	2.4 [1.3–5.7]	0.235
EOMES in Th9	14.9 [8.8–23.2]	12.9 [8.8–25.6]	0.939
EOMES in Th17	1.9 [1.1–3.5]	1.2 [0.8–2.0]	0.002
EOMES in Th17.1	17.1 [12.2–23.8]	17.6 [11.4–23.1]	0.825
EOMES in TEMRA	31.9 [17.4–49.5]	23.3 [12.1–43.7]	0.017
EOMES in TEM	25.3 [16.9–36.6]	21.4 [16.8–26.5]	0.001
EOMES in TCM	7.7 [5.3–10.2]	5.3 [4.2–7.1]	<0.001
EOMES in TNAIVE	3.5 [2.0–5.5]	1.5 [0.9–2.7]	<0.001
Validation cohort			
Th1 (% in CD4^+^T)	12.8 [10.6–15.5]	12.1 [10.3–15.6]	0.473
Th2 (% in CD4^+^T)	5.6 [4.0–9.2]	4.9 [4.4–7.0]	0.684
Th17 (% in CD4^+^T)	7.2 [5.1–9.7]	6.6 [5.0–8.6]	0.447
EOMES (% in CD4^+^T)	8.7 [6.1–11.8]	4.0 [3.1–6.4]	<0.001
EOMES in Th1	15.0 [8.2–21.5]	8.6 [5.7–13.9]	0.007
EOMES in Th2	2.4 [1.7–4.5]	1.3 [0.4–2.2]	0.001
EOMES in Th17	3.7 [2.1–6.8]	1.9 [1.1–3.8]	0.001

**FIGURE 1:**
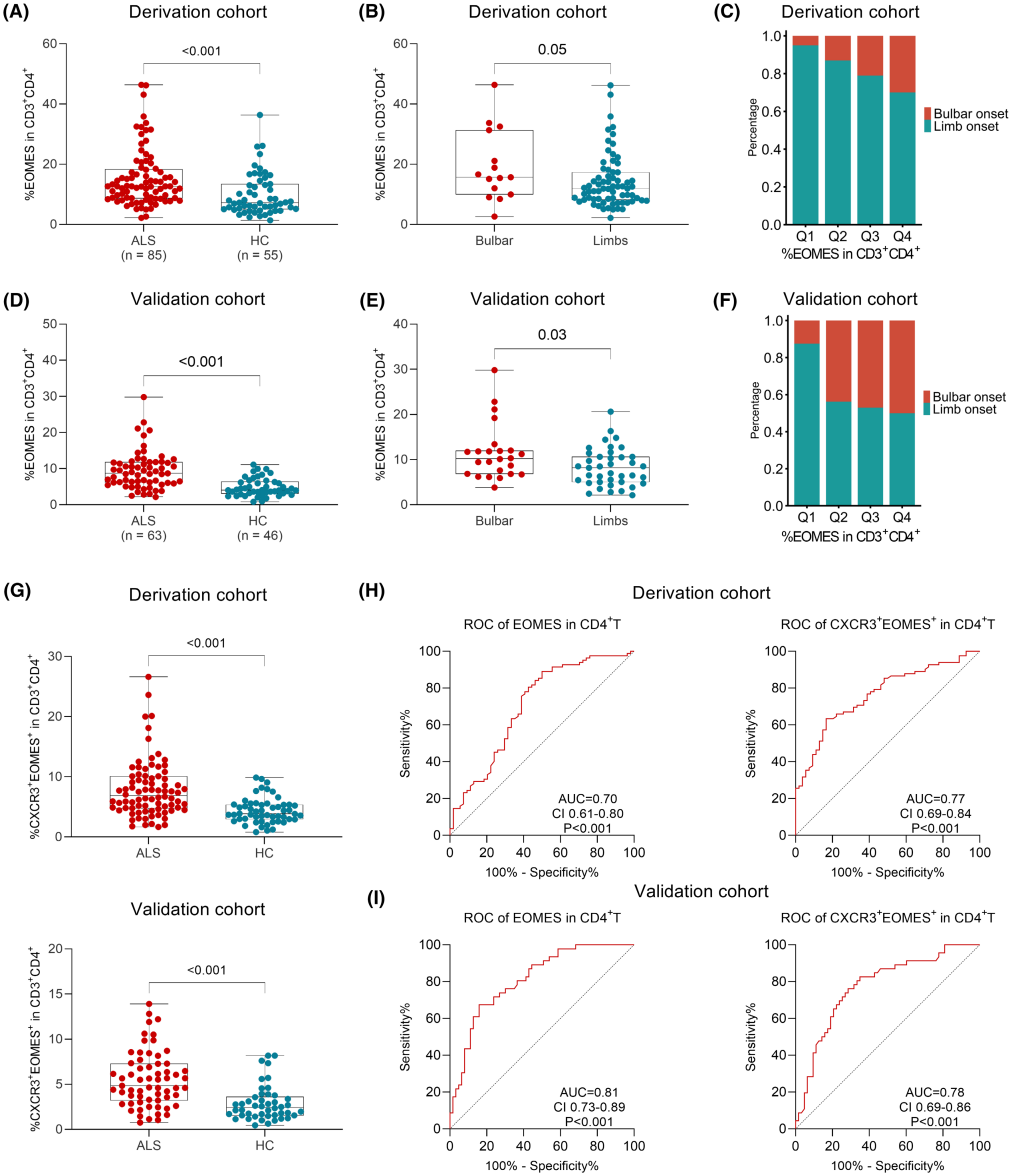
The EOMES^+^ expression in Th subsets in ALS and HC groups. (A) The EOMES expression was significantly elevated in ALS patients' CD4^+^T‐cells in the derivation cohort. (B) The EOMES expression was significantly higher in ALS patients with bulbar onset in the derivation cohort. (C) An increasing proportion of bulbar onset patients was observed among EOMES expression quarters in the derivation cohort. (D) The EOMES expression was significantly elevated in ALS patients' CD4^+^T‐cells in the validation cohort. (E) The EOMES expression was significantly higher in ALS patients with bulbar onset in the validation cohort. (F) An increasing proportion of bulbar onset patients was observed among EOMES expression quarters in the validation cohort. (G) The proportion of CXCR3^+^EOMES^+^ subset was elevated in ALS patients in both cohorts. (H) Receiver operating characteristic (ROC) curves were generated to evaluate diagnosis abilities of the EOMES in CD4^+^T‐cells and CXCR3^+^EOMES^+^ Th subset in the derivation cohort. (I) ROC curves were generated to evaluate diagnosis abilities of the EOMES in CD4^+^T‐cells and CXCR3^+^EOMES^+^ Th subset in the validation cohort. ALS, amyotrophic lateral sclerosis; EOMES, eomesodermin; HC, healthy control.

Subsequently, we measured EOMES expression in the peripheral CD4^+^T‐cells from ALS patients and HCs. The proportions of CD4^+^EOMES^+^T were significantly increased in the ALS cohort compared with HCs (12.7 [IQR: 8.7–18.7] vs. 7.2 [IQR: 5.0–13.7], *p <* 0.001, Figure 1A and Table 2). Moreover, the EOMES expression was enriched in Th1, Th17.1, and Th9 subsets in ALS patients and HCs (Table 2). Meanwhile, the EOMES was preferentially expressed in effector memory subsets (TEMRA and TEM). Moreover, we noticed that for those in subgroups with bulbar onset, the patients have higher EOMES expression in CD4^+^T‐cells (15.7 [IQR: 10.0–31.3] vs. 11.9 [IQR: 8.2–17.3], *p* = 0.05, Figure 1B). We then divided the patients into four quarters by EOMES expression in CD4^+^T‐cells, and observed an increasing proportion of bulbar onset patients among quarters (Figure 1C). The EOMES expression did not differ between genders.

The EOMES expression in the validation cohort was significantly increased as well, in consistency with the derivation cohort (8.7 [IQR: 6.1–11.8] vs. 4.0 [IQR: 3.1–6.4], *p* < 0.001, Figure 1D and Table 2). Similarly, the EOMES expression was higher in subgroups with bulbar onset (10.2 [IQR: 6.8–12.0] vs. 8.2 [IQR: 5.0–10.6], *p* = 0.03, Figure 1E,F). The EOMES expression in Th1 and Th17 was higher in ALS patients (15.0 [IQR: 8.2–21.5] vs. 8.6 [IQR: 5.7–13.9], *p* = 0.007, and 3.7 [IQR: 2.1–6.8] vs. 1.9 [IQR: 1.1–3.8], *p* = 0.001, respectively). However, we did not observe an elevated Th1 population in the validation cohort.

### 3.3 Elevated CXCR3 expression in CD4^+^EOMES^+^ T‐cells differentiates ALS from HCs

The EOMES expression was enriched in Th1, Th17.1, and TEMRA and TEM subsets, which were gated mainly according to the higher expression of CXCR3 and the lower expression of CCR7 or CD27. We then compared the CXCR3^+^EOMES^+‐^ subset in CD4^+^T‐cells between ALS and HC groups. Increased CXCR3^+^EOMES^+^ subset proportions were observed in ALS patients (6.9 [IQR: 4.7–10.1] vs. 3.9 [IQR: 2.9–5.4], *p <* 0.001, Figure 1G). Similarly, the ALS patients shared higher CXCR3^+^EOMES^+^ subset proportions in the validation cohort (4.8 [IQR: 3.2–7.3] vs. 2.4 [IQR: 1.6–3.6], *p <* 0.001, Figure 1G).

Receiver operating characteristic curves were further calculated to assess the predictive performance of the above‐mentioned CD4^+^T subsets in the derivation cohort. As was shown in Table 3 and Figure 1H, the CXCR3^+^EOMES^+^ subset may be the most promising univariate predictor with the biggest AUC estimated at 0.77 (CI 0.69–0.84, *p <* 0.001), followed by the EOMES in TNAIVE, and the CD4^+^EOMES^+^ subsets. The cutoff fraction of the CXCR3^+^EOMES^+^ subset was 5.637, with a sensitivity estimated at 63.41% and a specificity estimated at 83.33%. Similarly, the ROC curves of CD4^+^EOMES^+^T‐cells and the CXCR3^+^EOMES^+^ subset were further calculated in the validation cohort. The AUC of CD4^+^EOMES^+^T‐cells was estimated at 0.81 (CI: 0.73–0.89, *p <* 0.001), while the AUC of CXCR3^+^EOMES^+^ subset was estimated at 0.78 (0.68–0.87, *p <* 0.001; Figure 1I).

**TABLE 3:** Cutoff, sensitivity, and specificity among subsets in the derivation cohort.

Subsets	Sensitivity%	Specificity%	Cutoff	AUC	*p*
Th1	80.49	51.85	>9.185	0.67	0.001
EOMES in CD4^+^T	89.02	50.00	>7.130	0.70	<0.001
EOMES in Th1	43.90	81.48	>27.800	0.63	0.010
EOMES in Th17	58.54	72.22	>1.690	0.65	0.003
EOMES in TEMRA	76.83	42.59	>17.100	0.61	0.03
EOMES in TEM	39.02	85.19	>32.900	0.66	0.001
EOMES in TCM	56.10	77.78	>7.300	0.68	<0.001
EOMES in TNAIVE	54.88	85.19	>3.240	0.74	<0.001
CXCR3^+^EOMES^+^	63.41	83.33	>5.637	0.77	<0.001

Then, we compared the patients within 6 months of onsets. In the derivation cohort, 19 ALS patients' onsets were within 6 months. The median CD4^+^EOMES^+^ proportion was 13.0 (IQR 9.7–19.4) (Figure S2A). The median CXCR3^+^EOMES^+^ proportion was 7.7 (IQR 5.1–9.8) (Figure S2B). In the validation cohort, 16 ALS patients' onsets were within 6 months. The median CD4^+^EOMES^+^ proportion was 9.5 (IQR 5.3–12.0) (Figure S2C). The median CXCR3^+^EOMES^+^ proportion was 5.7 (IQR 3.3–7.9) (Figure S2D). ROC curve analyses revealed similar predictive performance with the prediction using whole cohorts (Figure S2E–H).

### 3.4 EOMES^+^ subsets were correlated with serum NFL levels

**FIGURE 2:**
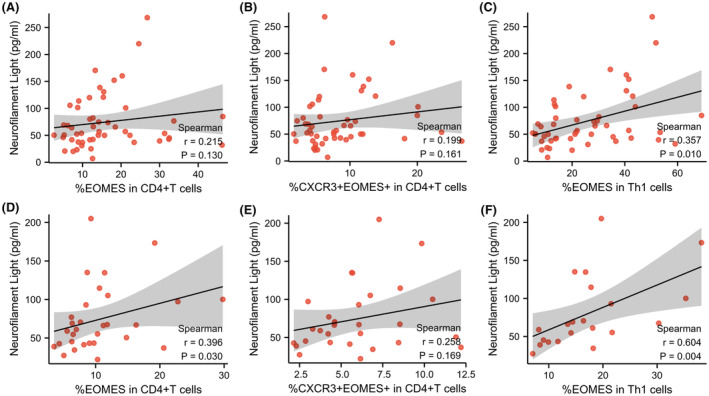
The correlations between serum NFL and EOMES^+^ Th subsets. (A–C) The correlations between serum NFL and EOMES subsets in the derivation cohort. (D–F) The correlations between serum NFL and EOMES subsets in the validation cohort. EOMES, eomesodermin; NFL, neurofilament light chain.

Both CD4^+^T subsets and EOMES‐positive subsets were not correlated with the decreased rate of the ALSFRS‐R scores. We assumed that the clinical scale might not be sensitive enough. Thus, we further quantified the patients' serum NFL level to evaluate subclinical motor neuron damage. In line with the previous studies, the NFL levels of the ALS patients were negatively correlated with disease duration and positively correlated with the decreased rate of ALSFRS‐R scores per month (*ρ* = −0.492, *p <* 0.001 and *ρ* = 0.494, *p <* 0.001). The direct linear correlations were not significant among subsets. Previous longitudinal studies have demonstrated that the blood NFL will dynamically increase in the early stage of the disease course (first 6–20 months) and remain relatively stable over times.^25^, ^26^ Thus, we selected ALS patients with a disease duration of more than 12 months. We observed correlations between EOMES subsets and serum NFL levels. The EOMES^+^ and CXCR3^+^EOMES^+^ subsets showed a tendency of positive correlation with serum NFL levels (*ρ* = 0.215, *p* = 0.130, and *ρ* = 0.199, *p* = 0.161, respectively. Figure 2A,B). More importantly, the EOMES in the Th1 cell subset was positively correlated with serum NFL levels in the derivation cohort (*ρ* = 0.357, *p* = 0.010, Figure 2C). In the validation cohort, we noticed the EOMES^+^ subset and the EOMES in the Th1 cell subset were both positively correlated with serum NFL levels (*ρ* = 0.396, *p* = 0.030, and *ρ* = 0.604, *p* = 0.004, respectively. Figure 2D,F). The CXCR3^+^EOMES^+^ subset also showed a tendency of positive correlation with serum NFL levels (*ρ* = 0.258, *p* = 0.169, Figure 2E), though without significance.

### 3.5 EOMES expression was correlated with ALS patients' survival

**FIGURE 3:**
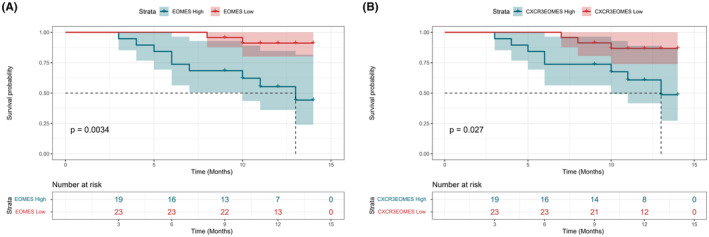
The survival curve analyses in the longitudinal study. (A) ALS patients with higher baseline median CD4^+^EOMES^+^T proportions were associated with worse prognosis. (B) ALS patients with higher baseline median CD4^+^CXCR3^+^EOMES^+^T proportions were associated with worse prognosis. ALS, amyotrophic lateral sclerosis; EOMES, eomesodermin.

To explore the relation between the EOMES expression and the prognosis of ALS, we recorded the ALSFRS‐R scores and primary endpoint events in the validation cohort recruited from Fujian Medical University Union Hospital in the following year. A total of 45 ALS patients were followed. Except for four patients dropped, 41 patients received follow‐up either in the clinic or by telephone. The most extended follow‐up period was 14 months. During the follow‐up period, 9 of 45 ALS patients reported endpoint events. At the 1‐year revisit, the average decrease of ALSFRS‐R score was 8 (IQR: 3.0–13.5), with an average progression rate of 0.3 (IQR: 0.7–1.2). When dividing the patients into two groups according to the baseline median proportion of CD4^+^EOMES^+^T‐cells, we noticed that ALS patients with higher EOMES expression had higher progression rates either (1.0 [IQR: 0.7–1.9] vs. 0.4 [IQR: 0.3–0.9], *p* = 0.010). Similarly, the Kaplan–Meier analysis revealed that ALS patients with higher EOMES or CXCR3^+^EOMES expression in CD4^+^T‐cells developed more endpoint events (Figure 3). Unfortunately, COX analysis adjusted for age, gender, disease duration, and site of onset did not reveal statistical significance (Table S1).

## 4 DISCUSSION

In this study, we performed in‐depth peripheral CD4^+^T profiling in two independent cohorts of ALS patients and corresponding matched healthy controls using multicolor flow cytometry. For the validation cohort, we longitudinally followed the ALS patients recruited from Fujian Medical University Union Hospital. We found the CD3^+^ and CD4^+^T‐cell counts were significantly decreased in ALS patients. The decreased counts of CD4^+^T‐cells and their role in ALS have been indicated in previous studies since it was linked to the deterioration of neurological function in both ALS patients and SOD1^G93A^ mice.^3^, ^4^, ^5^, ^9^, ^10^ The immunophenotyping of the conventional CD4^+^T subsets revealed increased Th1 proportions in ALS patients. Though without significance, the TEMRA and TEM proportions seemed to be increased, while the TCM proportions seemed to be decreased. The immune profile shifting toward a Th1/Th17 cell‐mediated pro‐inflammatory immune response in ALS patients has been reported recently.^27^ Moreover, the IL‐18 and IL‐1β, pro‐inflammatory cytokines closely linked to Th1 differentiation, were found elevated in the serum, CSF, and even muscles of ALS patients.^28^, ^29^, ^30^, ^31^ In addition, a significant augmentation of a Th1‐associated transcription factor, T‐bet, was revealed in the lumbar cord of SOD1^G93A^ mice at the mRNA level.^32^ Here, our results further enhanced the former evidence. Besides, we noticed an unexpected elevation of TEMRA and TEM proportions in ALS patients. Human TEMRA and TEM subsets are memory cells characterized by rapid effector function, containing Th1, Th2, and cytotoxic T lymphocyte (CTL) cells. A higher ratio of CD45RO/RA expression has been revealed in the PBMCs of ALS twins, suggesting an increased central or effector memory T‐cell subset.^33^ Moreover, a recent population‐based longitudinal cohort study revealed that the proportion of TEMRA subset in CD4^+^T‐cells was increased after ALS diagnosis and associated with higher mortality risk.^11^ Here, in concordance with previous studies, we confirmed this increased memory T‐cell subset in ALS patients, and further emphasized the critical roles of Th1 and effector memory subsets in the immunopathological process of ALS.

Recently, a hot debate has emerged regarding the role of EOMES in human CD4^+^T‐cell differentiation.^34^ It has been reported that EOMES could favor the phenotype shift of Th17 cells toward non‐classic Th1 cells and plays a key role in T helper cell (Th) plasticity.^14^ It has been claimed in a previous animal study that CD4^+^EOMES^+^T‐cells were required for the augmentation of CNS inflammation on adoptive transfer and the induction of chronic stage in experimental autoimmune encephalomyelitis mice.^16^ Recently, such a close association between the increased CD4^+^EOMES^+^T‐cells and the progression of the disease has been further verified in a cohort of 105 SPMS patients^17^ and even multiple neurodegeneration mouse models.^35^ The neurodegenerative mechanisms were believed to contribute a lot to the pathogenesis of SPMS. Thus, a similar role of peripheral EOMES expression in neurodegenerative disorders seemed to be reasonable. Here, in our study, we identified that the proportions of CD4^+^EOMES^+^T‐cells were significantly increased in ALS patients. Moreover, we found the EOMES expression was enriched in Th1, the TEMRA, and TEM subsets, regardless of ALS or HC groups. Considering the role of EOMES expression in CD4^+^T‐cell differentiation and the increased memory T‐cell subset, we noticed that in the immunophenotyping of CD4^+^T‐cells, we assumed a possible role of EOMES in the peripheral immunopathology in ALS patients.

Since the gating strategies of the T helper and effector memory subsets in the current study largely depended on the expression of several chemokine receptors, we wondered if unconventional EOMES‐positive subsets gated by these surface markers were more illustrative. Interestingly, we observed significantly increased CXCR3^+^EOMES^+^ subset proportions in the CD4^+^T‐cells of ALS patients. Our further ROC curve analysis indicated that the CXCR3^+^EOMES^+^ subset might be the most potent univariate predictor for ALS, with the biggest AUC estimated at 0.77. Upregulated migratory behavior resulting from increased CXCR3 expression has been demonstrated in peripheral lymphocytes from ALS patients.^36^ Considering our findings that the EOMES expression was enriched in Th1 and Th17.1 subsets, both were characterized by CXCR3 expression; we believed that EOMES, a transcription factor, might modulate the expression of surface chemokine receptor CXCR3, which might drive the cells to migrate toward CNS. However, further functional studies are needed to test our hypothesis.

As was indicated by Cui et al.,^11^ the lymphocyte subpopulations gated were not correlated with the decrease rate of the ALSFRS‐R. Same in our study, neither CD4^+^T subsets nor EOMES‐positive subsets were correlated with the decrease rate of the ALSFRS‐R scores. Thus, we further quantified the patients' sera NFL level to evaluate the motor neuron damage. Intriguingly, we noticed that multiple EOMES‐positive subsets were positively correlated with the serum NFL levels of patients' disease duration longer than 12 months, suggesting a potential relationship between the elevation of peripheral EOMES and the motor neuron damages. More importantly, we, for the first time, identified higher EOMES expression in peripheral CD4^+^T‐cells may be associated with worse prognosis in our validation cohort follow‐up. Our findings were potently supported by two recent reports. One recent animal study identified accumulated EOMES^+^ Th cells in the CNS of ALS and other neurodegenerative mouse models.^35^ Another study using human CSF identified high levels of effector T‐cells were enriched in the ALS patients' CSF, which was associated with poor survival. In their single‐cell transcriptomics analysis of CSF samples, clonally expanded CD4^+^ T‐cells were characterized by increased EOMES expression.^18^ Combining these findings, we believed that enriched EOMES in CD4^+^T‐cells, especially in CXCR3‐positive subsets, might represent a specific form of T‐cells that might migrate into the CNS and further deteriorate the inflammation environment in ALS patients. In this process, the elevated EOMES expression might be a critical hallmark for ALS patients.

A strength of our study is the relatively large sample sizes and three‐stage design compared to previous studies. However, the median ALSFRS‐R score in our ALS group was 41 with a median disease duration of 12 months, which represented a group of patients at an early stage. Thus, further studies are needed to see whether the changes in CD4^+^T subsets could be safely and smoothly fitted in patients at the later stages. Another limitation of our study is the discrepancy in bulbar onset patients' number between two stages. In addition, although part of our results is consistent with previous studies, there was a possible reverse causality between the changes in CD4^+^T subsets and CNS inflammation. Thus, a longitudinal cohort study with in‐depth immunophenotyping would be helpful to further elucidate the role of CD4^+^T subset changes. Besides, an investigation involving the CNS‐infiltrated CD4^+^ EOMES^+^T‐cells in ALS patients might be helpful.

In summary, we demonstrated altered peripheral CD4^+^T subsets and increased EOMES expression in ALS patients. We further demonstrated their associations with disease progression and prognosis. Identifying these associations in ALS may contribute to a better description of the immunopathological features of ALS. Besides, further understanding of the changes in CD4^+^T subsets and the critical role of EOMES expression may contribute to the development of potential therapeutics.

## AUTHOR CONTRIBUTIONS

SC, YC, and Z‐YZ contributed to the conception and design of the study; XH and C‐ZX contributed to the acquisition and analysis of data in the validation cohort; SC, XH, C‐BZ, KQ, YD, YW, C‐YL, H‐PH, YC, and Z‐YZ contributed to the acquisition and analysis of data; SC, XH, H‐HZ, and X‐YZ contributed to the interpretation of the data and statistical analysis; SC and S‐SL contributed to the drafting the text and preparing the figures. YC and Z‐YZ critically revised the manuscript.

## CONFLICT OF INTEREST STATEMENT

The authors declare no competing interests.

## Supporting information


Figure S1.

Figure S2.

Table S1.

## Data Availability

The data that support the findings of this study are available on request from the corresponding author. The data are not publicly available due to privacy or ethical restrictions.
