# Supplementary figures and images for: Eomesodermin expression in CD4+T‐cells associated with disease progression in amyotrophic lateral sclerosis
Source: CNS Neurosci Ther. 2023 Oct 18;30(4):e14503. doi: 10.1111/cns.14503 (PMC11017423; doi:10.1111/cns.14503)

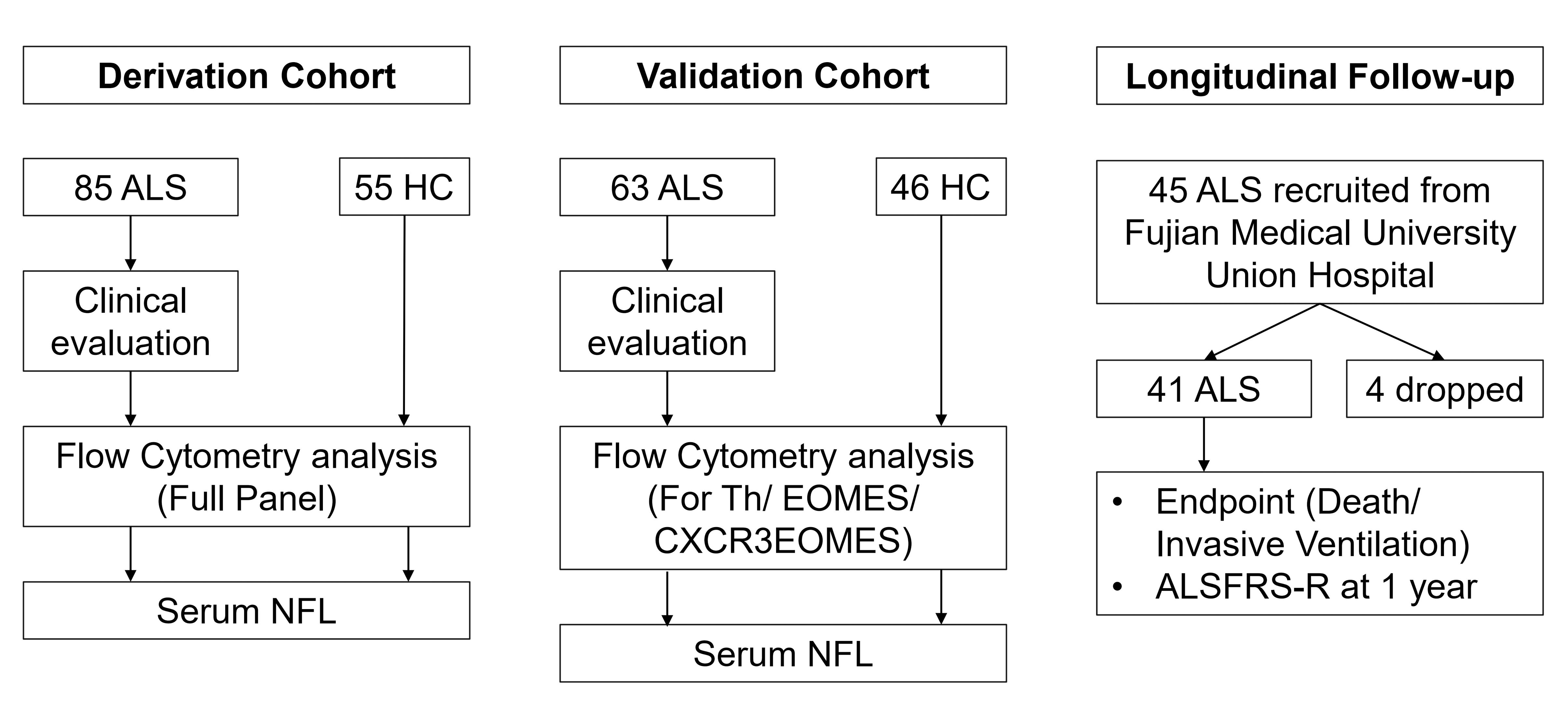

Supplement: Supplementary file 1 — Figure S1. [file CNS-30-e14503-s001.tif]

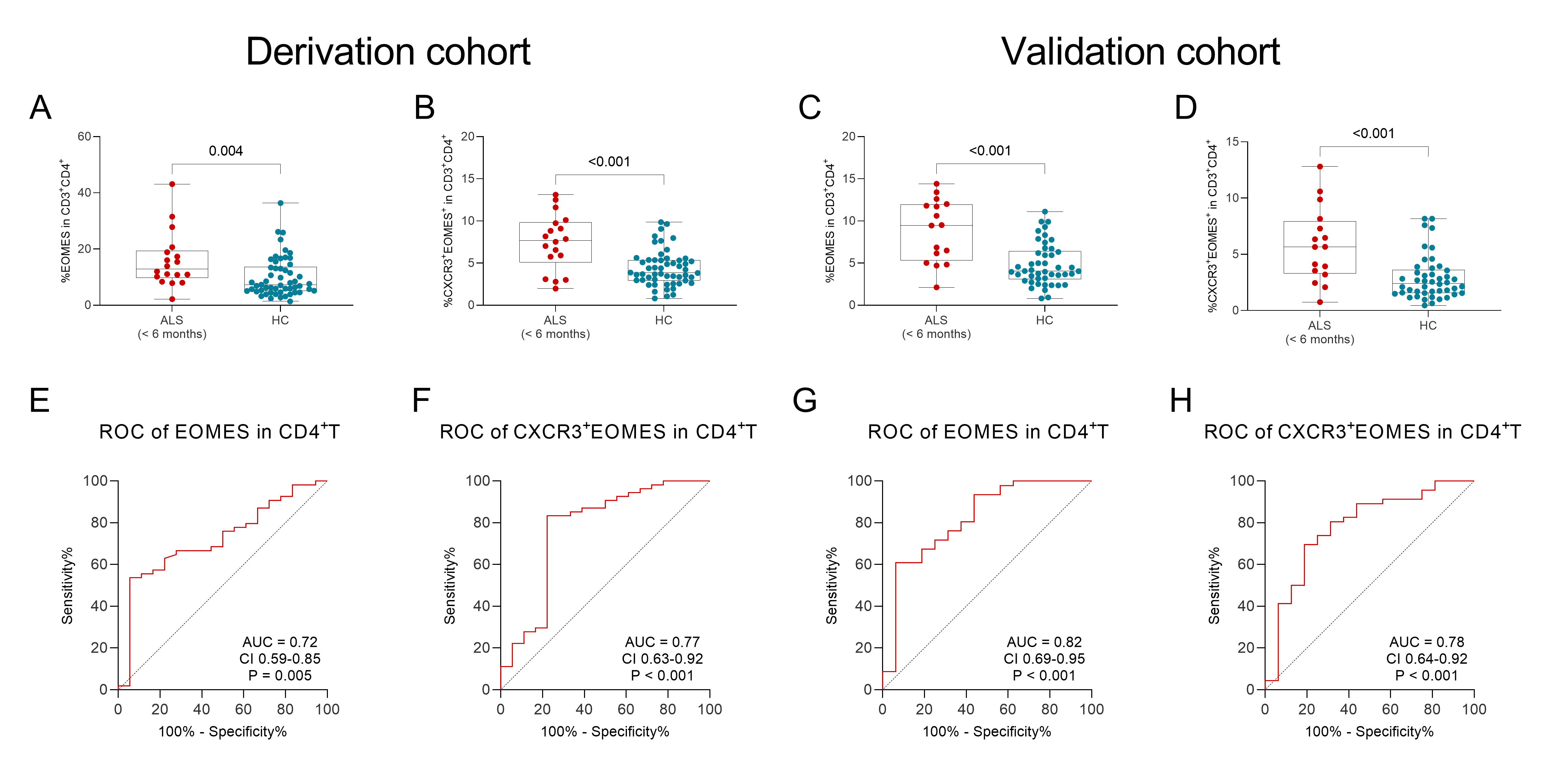

Supplement: Supplementary file 2 — Figure S2. [file CNS-30-e14503-s003.tif]
