# Supplementary material for: Eomesodermin expression in CD4+T‐cells associated with disease progression in amyotrophic lateral sclerosis
Source: CNS Neurosci Ther. 2023 Oct 18;30(4):e14503. doi: 10.1111/cns.14503 (PMC11017423; doi:10.1111/cns.14503)
Supplement: Supplementary file 3 — Table S1. [file CNS-30-e14503-s002.docx]

| **Supplemental table 1. Cox regression analyses for longitudinal follow-up.** | | | | | |
| --- | --- | --- | --- | --- | --- |
| Type | HR（95%CI） | *P*-value | FDR1^*^ | FDR2^**^ | FDR3^***^ |
| CD4 | 1.03 (0.96-1.10) | 0.438 | 0.646 | 0.584 | 0.548 |
| EOMES | 1.04 (0.95-1.14) | 0.367 | 0.551 | 0.489 | 0.459 |
| CXCR3^+^EOMES^+^ | 1.23 (0.98-1.54) | 0.080 | 0.120 | 0.107 | 0.133 |
| Th1 | 1.05 (0.92-1.21) | 0.467 | 0.646 | 0.623 | 0.583 |
| Th2 | 1.18 (0.97-1.44) | 0.102 | 0.153 | 0.136 | 0.170 |
| Th9 | 1.05 (0.97-1.14) | 0.216 | 0.324 | 0.288 | 0.318 |
| Th17 | 1.18 (1.01-1.38) | **0.039** | 0.058 | 0.052 | 0.065 |
| Th17.1 | 1.05 (0.95-1.17) | 0.342 | 0.513 | 0.456 | 0.428 |

^*^FDR1：Adjusted for age and gender.

^**^FDR2：Adjusted for age, gender, and disease duration.

^***^FDR3：Adjusted for age, gender, disease duration, and site of onset.

**Figure** **S1.** The flowchart of the study design.

**Figure** **S2.** The comparison and ROC analyses among ALS patients within 6 months of onset.
